# Carboxylic Acid Functionalization at the *Meso*-Position of the Bodipy Core and Its Influence on Photovoltaic Performance

**DOI:** 10.3390/nano9101346

**Published:** 2019-09-20

**Authors:** Filip Ambroz, Joanna L. Donnelly, Jonathan D. Wilden, Thomas J. Macdonald, Ivan P. Parkin

**Affiliations:** Department of Chemistry, University College London 20 Gordon St., London WC1H 0AJ, UK; filip.ambroz.16@ucl.ac.uk (F.A.); joanna.donnelly.15@ucl.ac.uk (J.L.D.); j.wilden@ucl.ac.uk (J.D.W.)

**Keywords:** Dye-sensitized solar cells, bodipy dye, co-sensitization, N719 dye, photoelectrochemical cells

## Abstract

Two bodipy dyes with different carboxylic acids on the *meso*-position of the bodipy core were prepared and used to sensitize TiO_2_ photoelectrodes. On the basis of spectroscopic characterization, the photoelectrodes were used to fabricate photoelectrochemical cells (PECs) for solar light harvesting. Photovoltaic measurements showed that both bodipy dyes successfully sensitized PECs with short-circuit current densities (*J*_SC_) two-fold higher compared to the control. The increase in generated current was attributed to the gain in spectral absorbance due to the presence of bodipy. Finally, the influence of co-sensitization of bodipy and N719 dye was also investigated and photovoltaic device performance discussed.

## 1. Introduction

Over the last few decades numerous types of photoelectrochemical cells (PECs) that convert sunlight to electricity have been extensively explored as an alternative photovoltaic technology to silicon [1,2]. The most promising include, dye-sensitized solar cells (DSSCs) [3,4], metal chalcogenide solar cells [5,6], organic/polymer solar cells [7,8,9], and perovskite solar cells [10,11]. While significant research has been conducted for their progress, they all have drawbacks in certain aspects that hinder large scale market employment [12]. For DSSCs in particular, the main efforts have been related to improving cell efficiencies where investigations have focused on two main components, such as the dye sensitizer and semiconducting electrode [13,14]. The foremost step in achieving high efficiencies is the light absorption by the dye molecules that should ideally cover as much of the visible spectrum as possible. To complement this, ruthenium-based dyes such as Di-tetrabutylammonium cis-bis(isothiocyanato)bis(2,2′-bipyridyl-4,4′-dicarboxylato)ruthenium(II) (best known as N719) and other ruthenium-based derivatives have been the most successful candidates thus far [15,16,17]. While N719 dye has been extensively used in DSSCs research since it shows promise of high efficiency, the limited supply, fluctuation in price, and environmental impact of ruthenium have all prompted investigations into other alternatives [18] Possible choices to replace ruthenium-based dyes include porphyrin [19,20], squaraine [21,22], and boron dipyrromethene (bodipy) dye complexes [23,24]. As a result of many unique features that bodipy dyes possess, they have emerged as an attractive class of functional dyes that can be used for a variety of applications [25,26]. They are known to exhibit characteristics such as ease of structural modification, minimum triplet state formation, photostability, low rates of intersystem crossing, and more [27], which renders them promising for use in the future [28]. In the past decade, a variety of bodipy dyes have been synthesized for use in solar cell applications, however most attempts were not prosperous since the efficiencies were shown to be poor [29]. This was mostly a result of insufficient light harvesting since efforts to design a metal free organic dye that would achieve panchromatic absorption proved to be rather ambitious [30]. Therefore, bodipy dyes were separately engineered to cover a particular absorption spectral range and co-sensitized together in a PEC with the aim to obtain high efficiency [31]. In addition, structural modifications and functionalization of the bodipy core was also shown to have a notable influence on the photovoltaic performance of PECs. For instance, by replacing fluorine on the bodipy core with long alkoxy groups, losses due to charge recombination can be minimized resulting in efficiencies in the range of 5.75% as reported by Ziessel et al [32].

Herein, we report the details of the synthesis and characterization of two bodipy sensitizers with different carboxylic acids at the *meso*-position [29] of the bodipy core. In addition, spectroscopy results of bodipy-sensitized TiO_2_ photoelectrodes are discussed followed by PEC fabrication and characterization using simulated sunlight. Finally, the influence of bodipy and N719 dye co-sensitization on the performance of PECs is also investigated.

## 2. Experimental Section

### 2.1. Materials

All chemicals used in this study were obtained from Sigma Aldrich (St. Louis, MO, USA), Acros Organics (Geel, Belgium), Alfa Aesar (Haverhill, MA, USA) or Thermo Fischer Scientific (Waltham, MA, USA). They were used as received unless otherwise stated.

### 2.2. Synthesis of Bodipy Dye 1 and 2

2,4-dimethylpyrrole (0.120 mL, 1.20 mmol, 2 eq.) and glutaric or succinic anhydride (0.600 mmol, 1 eq.) were dissolved in dry dichloromethane (DCM, 10.0 mL) under argon. BF_3_·Et_2_O (0.500 mL, 4.00 mmol) and TEA (0.420 mL, 3.00 mmol) were added and the reaction was heated under reflux for five hours. The organic material was washed with water, dried over MgSO_4_, filtered and concentrated in vacuo before purification by flash column chromatography on silica gel; eluent: Hexane/EtOAc/AcOH:50/50/0 to 0/97/3. 

#### 2.2.1. Bodipy Dye 1

4-(5,5-difluoro-1,3,7,9-tetramethyl-5H-4l4,5l4-dipyrrolo[1,,2-c:2’,1’-f][1,3,2]diazaborinin-10-yl)butanoic acid (22.0 mg, 0.066 mmol, 11%).^1^H NMR (600.130 MHz, CDCl_3_) δ_H_ 6.06 (s, 2H, ArH), 2.82–2.78 (m, 2H, CH_2_), 2.57–2.54 (m, 2H, CH_2_), 2.52 (s, 6H, 2CH_3_), 2.43 (s, 6H, 2CH_3_), 2.04–1.99 (m, 2H, CH_2_) ppm; R_f_ (EtOAc) = 0.1 (stained with bromocresol green).

#### 2.2.2. Bodipy Dye 2

3-(5,5-difluoro-1,3,7,9-tetramethyl-5H-4l4,5l4-dipyrrolo[1,2-c:2’,1’-f][1,3,2]diazaborinin-10-yl)propanoic acid (27.0 mg, 0.084 mmol, 14%).^1^H NMR (600.130 MHz, CDCl_3_) δ_H_ 6.06 (s, 2H, ArH), 3.36–3.33 (m, 2H, CH_2_), 2.71–2.69 (m, 2H, CH_2_), 2.52 (s, 6H, 2CH_3_), 2.45 (s, 6H, 2CH_3_) ppm; R_f_ (EtOAc) = 0.1 (stained with bromocresol green).

### 2.3. Preparation of Photoelectrochemical Cells (PECs)

PEC fabrication followed the procedures already reported in our previous work [14]. Briefly, TiO_2_ photoelectrodes were prepared by doctor blading commercially available TiO_2_ paste (GreatCell Solar, DSL-18NRT, Queanbeyan, Australia) onto pre-cleaned fluorine-doped tin oxide (FTO) glass followed by sintering at 500 °C for 30 min which resulted in a uniform layer of anatase TiO_2_ nanoparticles (NPs) labelled as a transparent layer. On this layer, an additional layer of bigger-sized TiO_2_ NPs (WER2-0 paste, GreatCell Solar, Queanbeyan, Australia) was deposited (doctor blading technique) followed by another sintering at 500 °C for 30 min. The second layer was labelled as light-scattering layer. The photoelectrodes were then subject to treatment with an aqueous solution of titanium tetrachloride (TiCl_4_, 40 mM) for 30 min at ≈ 70 °C followed by another sintering at 500 °C for 30 min. It should be noted that the active solar cell area of the photoelectrodes was 0.1256 cm^2^. Control (unsensitized) PECs (see procedures below for dye sensitization), were directly eclipsed with counter electrodes (Pt-coated FTO glass, GreatCell Solar, Queanbeyan, Australia) with a thermoplastic sealant (GreatCell Solar, MS004610, Queanbeyan, Australia) following by heating at ≈ 100 °C for 10 min to seal the sealant. An electrolyte consisting of iodine (I_2_, 0.05 M), 1,2-dimethyl-3-propylimidazolium iodide (DMPII, 0.6 M), guanidium thiocyanate (0.10 M) and 4-tert-Butylpyridine (TBP, 0.5 M) in a mixture of acetonitrile and valeronitrile (volume ratio, 85:15) was injected between the sealed electrode via vacuum filling.

### 2.4. Dye Sensitization

Bodipy dye 1 and 2 sensitized photoelectrodes underwent dye sensitization with a solution of a relevant dye (0.5 mM) in acetonitrile for 20 h in the dark.

Control (sensitized only with N719) photoelectrodes underwent dye sensitization with a solution of ruthenizer 535-bisTBA (Solaronix 0.5 mM, Aubonne, Switzerland) in absolute ethanol for 20 h in the dark.

Bodipy dye 1 and 2 co-sensitized photoelectrodes underwent dye sensitization with a solution that was a mixture of bodipy dye 1 or 2 solution in absolute ethanol (0.5 mM)—5 vol% and a solution of ruthenizer 535-bisTBA (0.5 mM, Solaronix, Aubonne, Switzerland) in absolute ethanol for 20 h in the dark.

## 3. Characterisation

Monitoring of all reactions was achieved using 60 F254 silica coated aluminium TLC plates by Merck. Visualisation of these was carried out using UV light of wavelength of 254 and/or 365 nm. Purification was achieved by flash column chromatography using silica gel (43–60 µm) from Merck.

All ^1^H, ^13^C, ^11^B and ^19^F nuclear magnetic resonance spectra were recorded at 600 MHz using the Bruker Avance III 600 Cryo or at 700 MHz with the Bruker Avance Neo 700. Instruments clearly indicated for each spectra. Chemical shifts are reported in ppm relative to the internal standard TMS as follows: chemical shift, multiplicity (s = singlet, d = doublet, t = triplet, q = quartet, sep = septet, m = multiplet), coupling constant(s), integration, and assignment using either CDCl_3_ or DMSO-*d^6^* as a solvent and TMS as an internal standard.

X-Ray photoelectron spectroscopy (XPS) analysis was carried out using a Thermo Scientific K-alpha photoelectron spectrometer with monochromatic Al-K_α_ radiation. Peak positions were calibrated to carbon (284.8 eV) and plotted using the CasaXPS software. The measurements were performed on control and sensitized photoelectrodes. X-Ray diffraction (XRD) analysis was performed with a Bruker D8 discovery X-Ray diffractometer using monochromatic Cu K_α1_ and Cu K_α2_ radiation of wavelengths 1.54056 and 1.54439 Å, respectively.

The UV/Vis absorption spectra were taken on a Perkin Elmer Lambda 950 instrument with a measurement interval of 1 nm.

The photoluminescence (PL) spectra were recorded using Horiba FluoroMax-4 spectrofluorometer equipped with a PMT detector.

Fourier-transform infrared (FTIR) spectra were recorded with a Bruker FTIR Spectrometer Alpha II with an attenuated total reflection (ATR) attachment. Measurements were performed in the mid-IR region (4000–400 cm^−1^).

J–V measurements were performed under one-sun (AM 1.5G) illumination using a LOT calibrated solar simulator with a Xenon lamp. Devices were connected to a Keithley 2400 source meter to output the data. Photocurrent measurements were obtained with a halogen lamp chopped to a frequency of 188 Hz through a Newport monochromator; a 4-point probe in connection with a lock-in amplifier is used to collect data. The monochromatic beam is calibrated using a Silicon photo-diode.

## 4. Results and Discussion

### 4.1. Synthesis

**Scheme 1:**
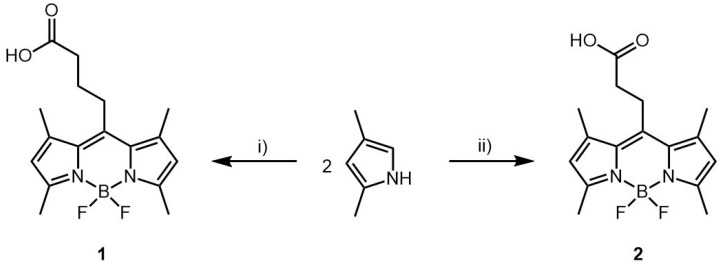
Synthetic routes towards bodipy dyes 1 and 2. (**1**) Succinic anhydride, dichloromethane (DCM), BF_3_·Et_2_O, reflux 5h, BF_3_·Et_2_O, TEA, 16 h. (**2**) Glutaric anhydride, DCM, BF_3_·Et_2_O, reflux 5h, BF_3_·Et_2_O, TEA, 16 h.

The synthesis of bodipy dye 1 and 2 is depicted in Scheme 1 and is based on previously reported synthetic strategies [33,34]. (See Supplementary Materials for more details) Molecular structures of the obtained compounds were characterized by spectroscopic methods, such as NMR which is shown in Supplementary Materials Figure S1 and S2. Singlet peaks integrating to two protons at 6.06 ppm for both compounds confirm the successful construction of the BODIPY scaffold.

### 4.2. Optical Properties

**Figure 1:**
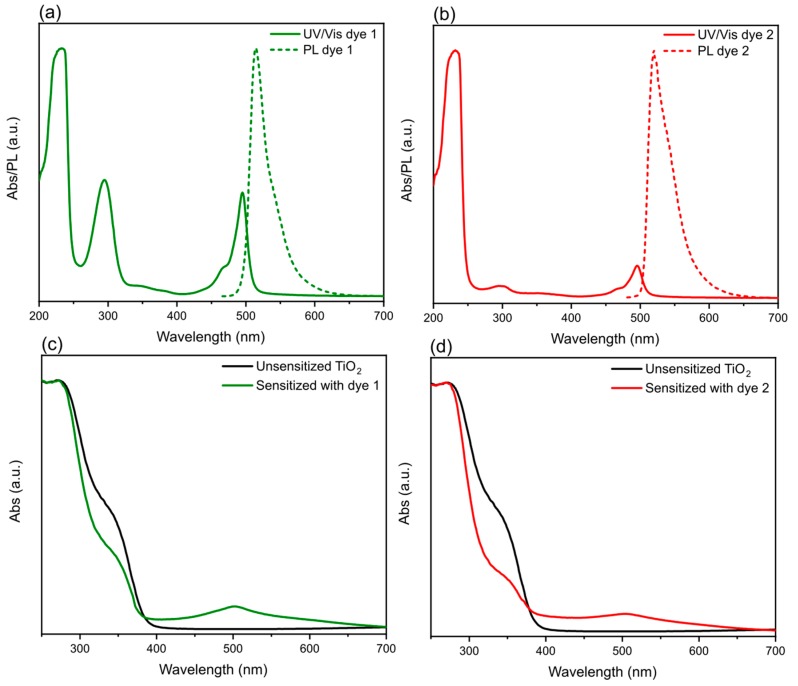
UV/Vis absorption (solid line) and photoluminescence (PL) emission (dotted lines) spectra of bodipy (**a**) dye 1 and (**b**) dye 2 solutions. (Excitation wavelength for PL emission was 400 nm) UV/Vis absorption spectra of TiO_2_ sensitized photoelectrodes of bodipy (**c**) dye 1 and (**d**) dye 2, respectively. Black lines represent untreated TiO_2_ photoelectrodes where green (dye 1) and red (dye 2) lines correspond to TiO_2_ sensitized photoelectrodes.

The absorption and PL emission spectra of solutions of bodipy dyes 1 and 2 are illustrated in Figure 1a,b. Both dyes exhibit three absorption features (more pronounced for dye 1 due to higher molar attenuation coefficients (ε) at those particular wavelengths—Table 1) with two located in the UV region and one in the visible (green) portion of the electromagnetic spectrum. From the absorption onset of the dyes, their band gap values were determined to be ≈ 2.4 eV (Table 1). Due to Stokes shift, where the position of the band maxima of the absorption was at ≈ 495 nm, the emission of the dyes resulted to be 514 nm for dye 1 and 520 nm for dye 2. The bodipy dyes were then used for PECs, by sensitizing TiO_2_ photoelectrodes in a dye solution and their corresponding UV/Vis spectra are shown in Figure 1c,d. To evaluate if the TiO_2_ photoelectrodes successfully absorbed the dyes, comparison was made to the untreated (unsensitized) TiO_2_ photoelectrodes. As can be seen in Figure 1c,d, both sensitized TiO_2_ photoelectrodes exhibited a broad absorption feature in the visible spectrum allowing the harvesting of more energy from photons. This can be attributed to the dye absorption (λ_max_ at 502 and 504 nm for bodipy dyes 1 and 2, respectively). Compared to the bodipy solutions, sensitized TiO_2_ photoelectrodes exhibited a bathochromic shift that occurred as a result of the interaction, between the dye and TiO_2_ nanoparticles (suppression of H-aggregation). [35] Moreover, to investigate the molecular packing of dye molecules [36] X-Ray diffraction (XRD) was also performed. Supplementary Materials Figure S4 shows the XRD diffraction patterns for unsensitized and sensitized TiO_2_ photoelectrodes where no difference between the samples was observed.

### 4.3. Spectroscopy Characterization

**Figure 2:**
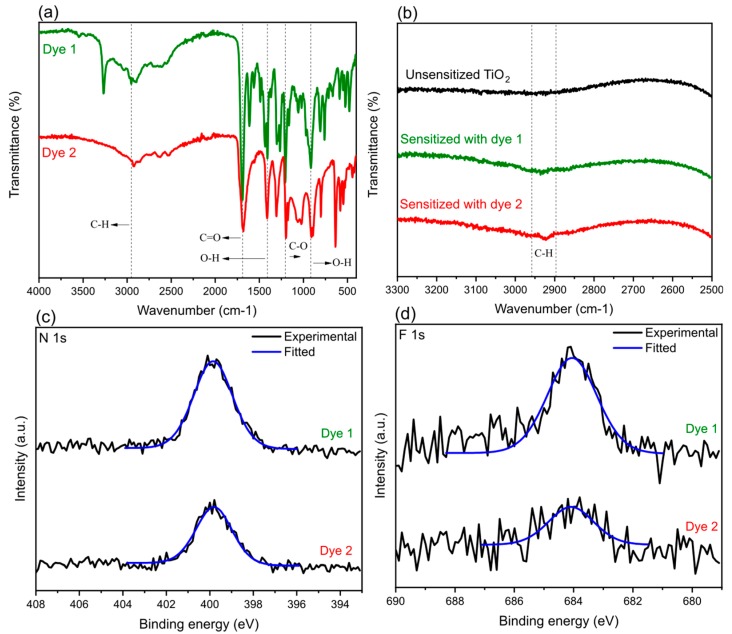
Fourier-transform infrared (FTIR) spectra of bodipy (**a**) dye 1 (green curve) and dye 2 (red curve) in a powder form along with the inset (**b**) of untreated (black curve) and sensitized TiO_2_ photoelectrodes on glass substrate. The whole range for the FTIR spectra of TiO_2_ photoelectrodes can be seen in Supplementary Materials Figure S5. The high-resolution (**c**) N 1s and (**d**) F 1s X-ray photoelectron spectroscopy (XPS) spectra for sensitized TiO_2_ photoelectrodes.

FTIR spectroscopy was used to obtain infrared spectra (% transmittance) of synthesized bodipy dyes which can be seen in Figure 2. A broad band in the region of around 3500–2500 cm^−1^ can be observed (Figure 2a) which is a typical characteristic for compounds containing a carboxyl group (O–H stretch) [38]. Since this band is in the same region as the C–H stretching bands, a slightly distorted absorption pattern is observed where the former band is superimposed on the sharp C–H stretching bands. Furthermore, a strong peak appeared around 1700 cm^−1^ for both dyes which was assigned to C=O stretching of the carbonyl group [35]. Additional peaks that were identified for both spectra were located in the regions of around 1400, 1200, and 910 cm^−1^ (see Supplementary Materials Table S1 for the exact values) and were assigned to O–H bend, C–O stretch, and O–H bend, respectively.

FTIR measurements were also performed with the intention to investigate interaction of individual dyes on sensitized TiO_2_ electrodes. However, our results showed that the substrates used for TiO_2_ deposition strongly absorbed below 2500 cm^−1^ which prevented characterization in this region (Supplementary Materials Figure S5). Despite this, by comparing regions of around 3000 cm^−1^ between sensitized and untreated TiO_2_ photoelectrodes, a slight increase in absorbance was observed for both sensitized TiO_2_ photoelectrodes. (Figure 2b) These bands were assigned to C–H stretching of the dye compounds.

X-Ray photoelectron spectroscopy (XPS) was carried out to identify relevant elements present on the surface of untreated and sensitized TiO_2_ photoelectrodes. Elements (N and F) associated to the compounds of the dyes were identified on both sensitized TiO_2_ photoelectrodes (Figure 2c) while only noise was observed in the same binding energy regions for the untreated TiO_2_ photoelectrode. (Figure S6). It is noteworthy that efforts related to the detection of boron resulted to be negative. However, such results are not surprising since it is notoriously difficult to detect B1’s peak due to its very low sensitivity.

### 4.4. Photoelectrochemical Properties

**Figure 3:**
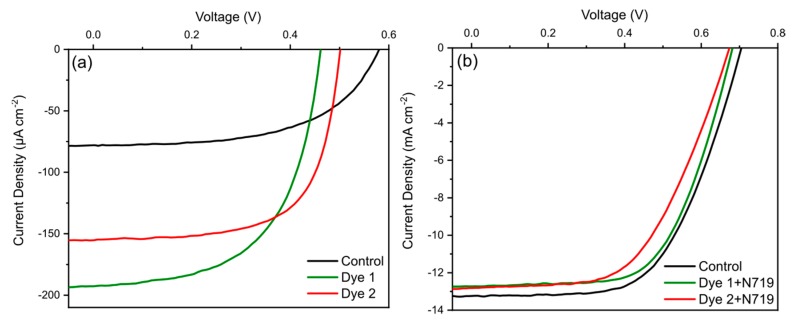
Photocurrent–voltage (*J–V*) characteristics of photoelectrochemical cells (PECs) sensitized only with (**a**) dye 1 and 2 including a control (unsensitized) together with (**b**) N719 co-sensitized. The control PEC in (**b**) was sensitized only with a dye N719.

Photoelectrochemical cells (PECs) were fabricated using bodipy dyes 1 and 2 as photosensitizers for nanocrystalline TiO_2_ photoelectrodes. Figure 3a shows the photocurrent–voltage (*J–V*) curves of PECs and the photovoltaic parameters are summarized in Table 2. Compared to control (unsensitized), PECs sensitized with bodipy dyes showed a significant increase in *J*_SC_ values (≈2.5-fold increase for dye 1 and ≈ 2-fold increase for dye 2). Such a phenomenon is primarily attributed to the improved light harvesting due to the presence of the bodipy dyes. Moreover, the fill factor (FF) also increased for sensitized PECs while V_OC_ decreased. The latter can be related to the changes in the band gap energy (E_g_) where an E_g_ decrease results in higher *J*_SC_ and lower V_OC_. Nevertheless, the overall power conversion efficiency (PCE) of bodipy sensitized PECs doubled compared to the control. On the other hand, the results indicate that the proximity of the carboxylic acid group to the bodipy core notably influenced the photovoltaic parameters for PECs. Specifically, PECs sensitized with a bodipy dye 1 (distance of three carbons) were shown to exhibit better *J*_SC_ as opposed to the bodipy dye 2 (distance of two carbons) sensitized PECs that had higher V_OC_ and FF. Nevertheless, the overall PCE was not influenced by such changes (Table 2). It is noteworthy that the average values of the photovoltaic parameters for different batches of devices, shown in Figure S7, did not show a change in the trends, as discussed herein. Furthermore, the values of PV parameters for bodipy sensitized PECs were low where one of the reasons together with the nature of the absorption onset of the dyes can be related to their aggregation on the TiO_2_ photoelectrodes, thus increasing charge recombination and reducing electron injection to the n-type layer of the photoelectrode [39,40].

Previous reports have shown that the performance of DSSCs containing co-adsorbents, bodipy and N719 can be improved compared to a single sensitizer [35]. Therefore, in an attempt to improve PV parameters bodipy dyes in this study were used and co-sensitized with N719 to investigate if the same conclusions can also be extended to the other combinations of sensitizers. In comparison with the control (sensitized only with N719), both *J*_SC_ and V_OC_ diminished for bodipy (dye 1 and 2) co-sensitized (5 vol%) [35] PECs (Figure 3b and Table 2). A particularly noticeable decrease was observed for V_OC_ where the parameter is influenced by the recombination rate and adsorption mode of the sensitizer [41] that when compared to the control, negatively influenced the co-sensitized PECs. Therefore, the bodipy dyes used in this study were shown to have a negative effect when implemented together with a N719 dye. We postulate that such an outcome was as a result of an increase in recombination centers which can be attributed to either a mismatch in energy levels or unsuitable molar absorption coefficient. Despite this, the bodipy dyes were shown to successfully sensitize TiO_2_ photoelectrodes, and further work into modifying the functional groups could lead to improved single sensitized, and co-sensitized PV performance. Specifically, efforts should be made into replacing electronegative elements, for example fluorine, with other functional groups that do not have such tendency to attract a bonding pair of electrons since this characteristic negatively affects the PV performance. In addition, other methods that aim to extend the absorption onset include attachments of an electron donor or acceptor to the C2 and C6 positions [29].

## 5. Conclusions

Two bodipy dyes with different carboxylic acids on the *meso*-position of the bodipy core have been synthesized. The bodipy dyes were used to sensitize TiO_2_ photoelectrodes which were characterized using spectroscopic techniques (UV/Vis absorption, FTIR and XPS). On the basis of these results, the TiO_2_ photoelectrodes were used to fabricate PECs. Their PV parameters were analyzed, and the results showed superior light harvesting and thus higher power conversion efficiencies compared to the control (unsensitized) PECs. Furthermore, the bodipy dyes were co-sensitized (5 vol%) with a N719 dye to investigate their interactions in a PEC. The results showed a decrease in efficiencies indicating co-sensitization had a negative effect on the photovoltaic performance parameters of co-sensitized PECs. Further improvements in efficiencies are possible by designing/modifying bodipy dyes with different light absorbing groups within the organic framework to tune the absorption spectral range with high molar extinction coefficients and use them as co-sensitizers for solar cell devices.

## Figures and Tables

**Table 1:** Summary of spectral properties of bodipy dye 1 and 2 in ethanol solution (0.5 mM) and on photoelectrodes. The band gap values (E_g_) were determined following the procedure reported elsewhere [37] and also from the Tauc plots (Supplementary Materials Figure S3) where the obtained values were comparable.

Sensitizer	Solution Abs λ_max_ (nm)	Solution PL λ_max_ (nm)	Photoelectrode Abs λ_max_ (nm)	E_g_ (eV)	ε (M^−1^ cm^−1^)
Dye 1	232, 295, 495	514	502	2.39	6124 (at 495 nm)
Dye 2	232, 295, 496	520	504	2.38	1350 (at 496 nm)

**Table 2:** Summary of photovoltaic parameters for different PECs sensitized with bodipy dye 1 or 2 and co-sensitized with dye 1 + N719 or dye 2 + N719 including the corresponding control (unsensitized and N719 sensitized) PECs.

PEC	*J*_SC_ (µA cm^−2^)	V_oc_ (mV)	FF (%)	PCE (%)
Control (unsensitized)	78.2	579.6	56.3	0.025
Dye 1	192.6	461.3	58.0	0.051
Dye 2	154.8	501.0	66.4	0.051
Control (N719 sensitized)	13.2	704.7	59.2	5.5
Dye 1 + N719	12.7	681.3	61.6	5.3
Dye 2 + N719	12.8	673.1	55.5	4.8

## Supplementary Materials

The following are available online at https://www.mdpi.com/2079-4991/9/10/1346/s1, Figure S1: ^1^H-NMR of bodipy dye 1, Figure S2: ^1^H-NMR of bodipy dye 2, Figure S3: Tauc plots for solutions of bodipy (a) dye 1 and (b) dye 2, Figure S4: XRD patterns for TiO_2_ photoelectrodes, Figure S5: FTIR spectra of untreated and sensitized TiO2 photoelectrodes, Figure S6: The high-resolution (a) N 1s and (b) F 1s XPS spectra for control (unsensitized) TiO2 photoelectrode, Figure S7: Average (a) *J*_SC_, (b) V_OC_, (c) FF and (d) power conversion efficiency (PCE) of the fabricated PECs sensitized only with bodipy dye 1 or 2 including a control (unsensitized), Table S1: FTIR peak wavenumbers and assignments for bodipy dye 1 and 2.
