# Supplementary material for: Carboxylic Acid Functionalization at the Meso-Position of the Bodipy Core and Its Influence on Photovoltaic Performance
Source: Nanomaterials (Basel). 2019 Sep 20;9(10):1346. doi: 10.3390/nano9101346 (PMC6835471; doi:10.3390/nano9101346)
Supplement: Supplementary file 1 [file nanomaterials-09-01346-s001.pdf]

Supplementary Materials

Carboxylic Acid Functionalization at the Meso-Position of the Bodipy Core and Its Influence on Photovoltaic Performance

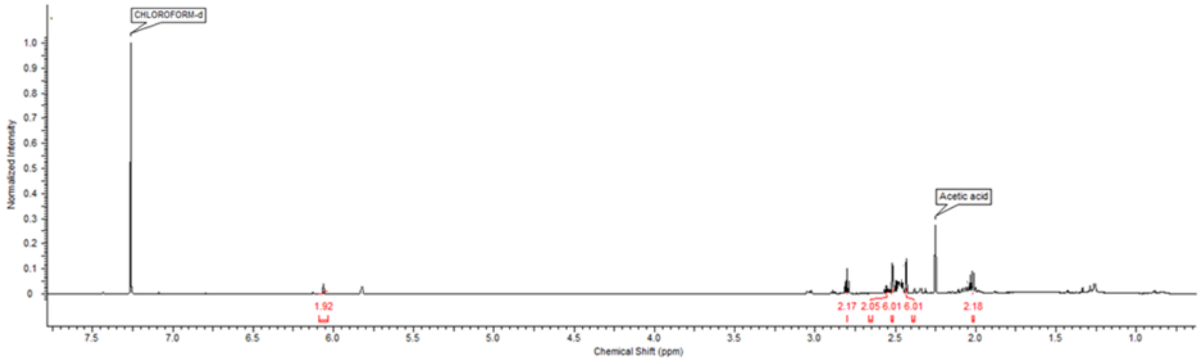

Figure S1. ^{1}H-NMR of bodipy dye 1.

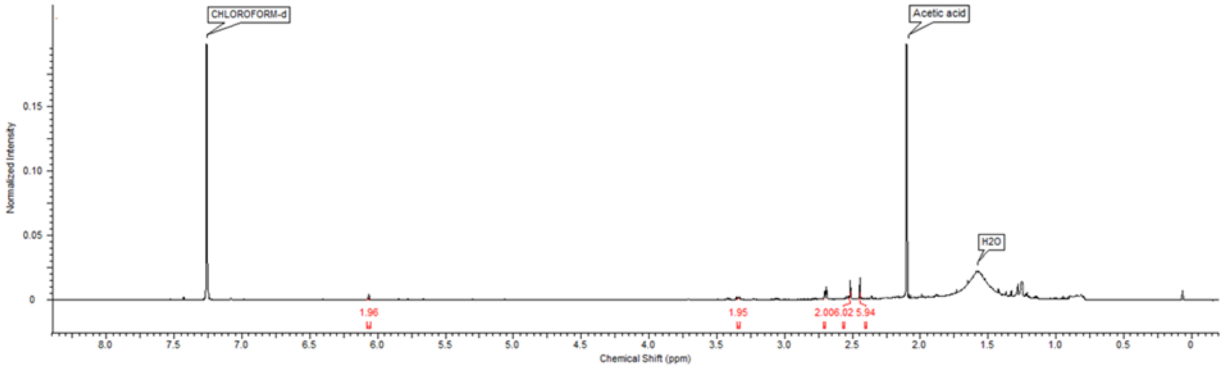

Figure S2. ^{1}H-NMR of bodipy dye 2.

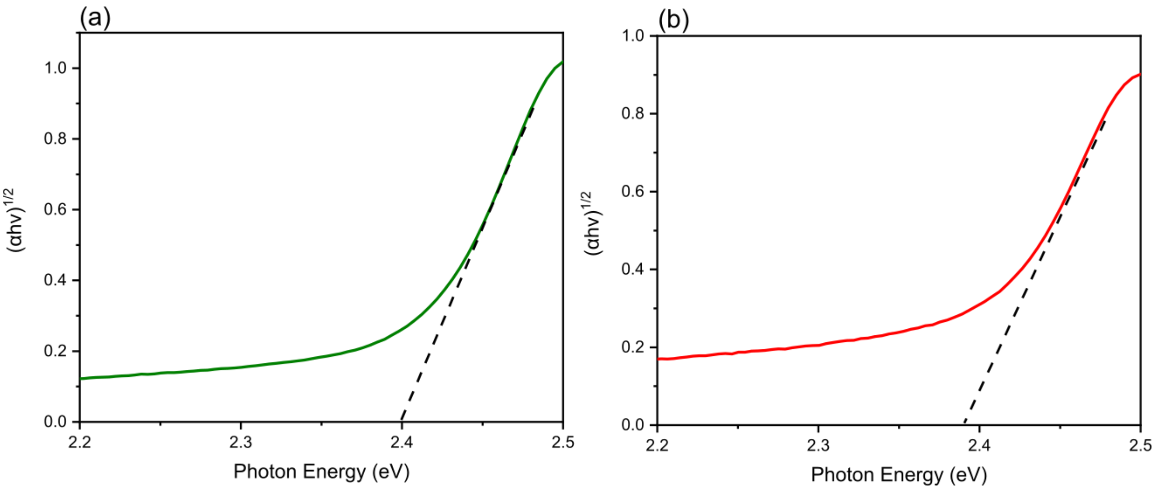

Figure S3. Tauc plots for solutions of bodipy (**a**) dye 1 and (**b**) dye 2.

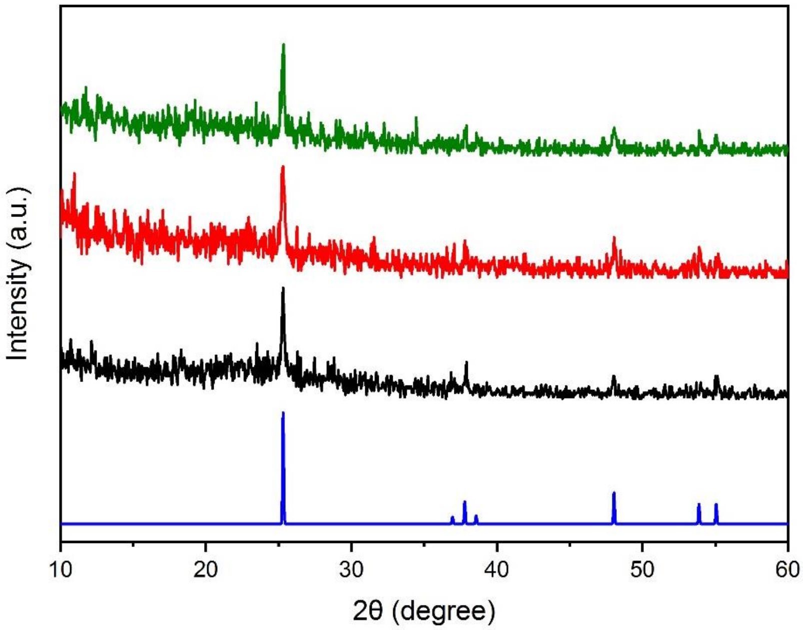

Figure S4. XRD patterns for TiO_2 photoelectrodes that were sensitized with bodipy dye 1 (green) and bodipy dye 2 (red) together with the unsensitized TiO_2 photoelectrode (black). Standard anatase TiO_2 phase is shown in blue (bottom).

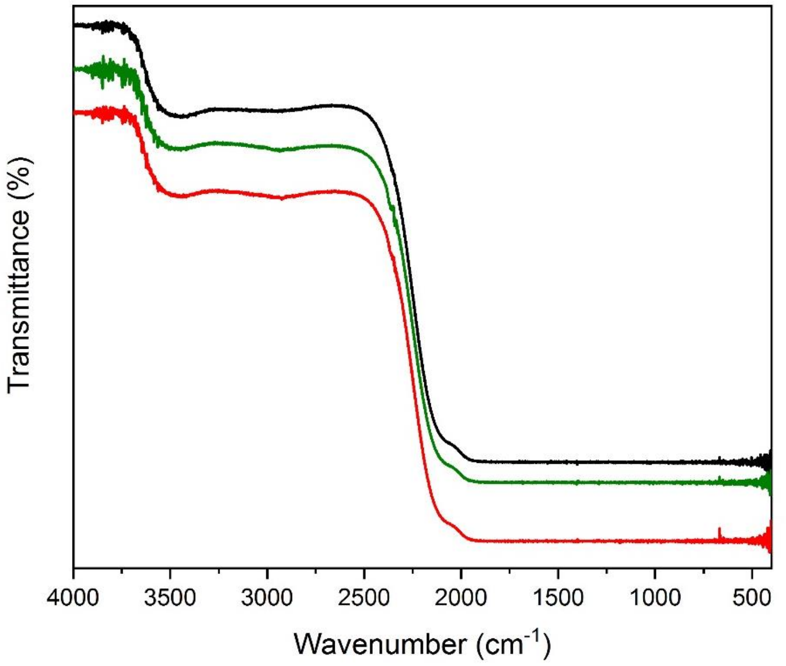

Figure S5. FTIR spectra of untreated (black curve) and sensitized TiO_2 photoelectrodes (green curve represent dye 1 while red curve dye 2).

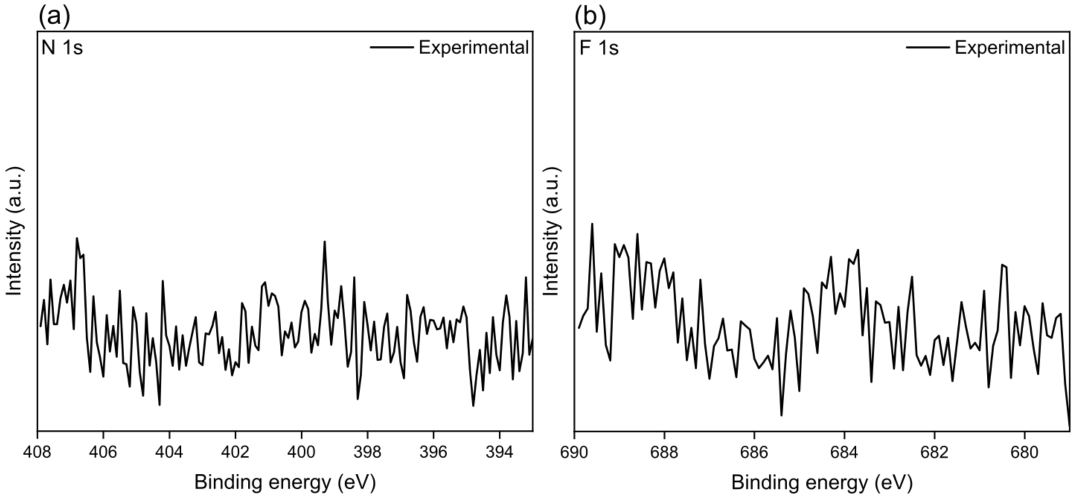

Figure S6. The high-resolution (**a**) N 1s and (**b**) F 1s XPS spectra for control (unsensitized) TiO_2 photoelectrode.

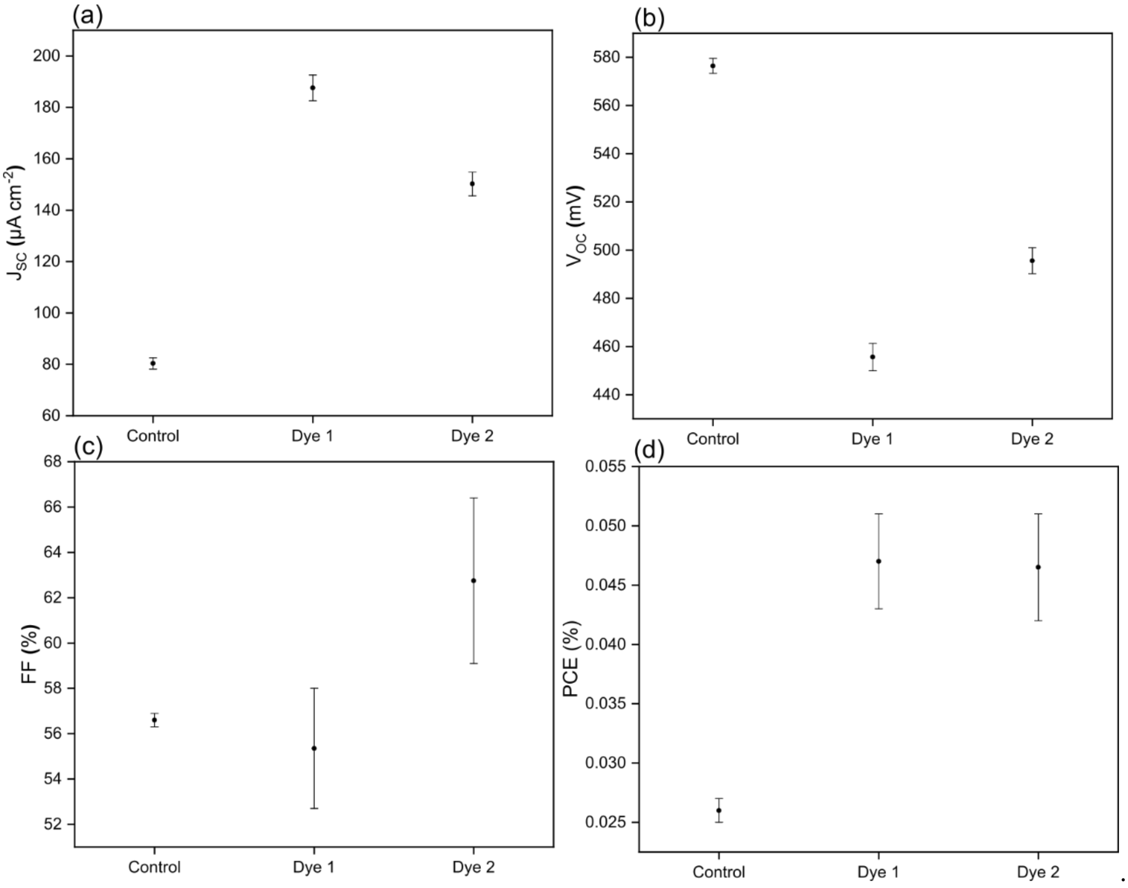

Figure S7. Average (**a**) J_{SC}, (**b**) V_{OC}, (**c**) FF and (**d**) power conversion efficiency (PCE) of the fabricated PECs sensitized only with bodipy dye 1 or 2 including a control (unsensitized). The averages were calculated over 2 separate batches of devices and the standard error has been illustrated as error bars.

Table S1. FTIR peak wavenumbers and assignments for bodipy dye 1 and 2. The symbols (ν and δ) refer to stretching or bending, respectively.

Dye 1 wavenumber (cm^{-1})	2953	1691	1407	1205	915
Dye 2 wavenumber (cm^{-1})	2927	1680	1410	1197	909
Assignment	ν(C-H)	ν(C=O)	δ(O-H)	ν(C-O)	δ(O-H)
